# U-shaped association between untreated caries and body mass index in adults at Rabat dental University hospital, Morocco: cross sectional study

**DOI:** 10.1186/s13104-016-2356-0

**Published:** 2017-01-03

**Authors:** Sanaa Chala, Manal El Aidouni, Redouane Abouqal, Faïza Abdallaoui

**Affiliations:** 1 Research Team on Oral Ecosystem, Department of Endodontics and Restorative Dentistry, Faculty of Dental Medicine, Laboratory of Clinical and Epidemiological Research, Mohammed V University in Rabat, 6212 Rabat, Morocco; 2 Laboratory of Clinical and Epidemiological Research. Faculty of Medicine and Pharmacy, Mohammed V University in Rabat, Rabat, Morocco; 3 Mohammed V Military Teaching Hospital, Rabat, Morocco

**Keywords:** High body mass index, Low body mass index, Caries severity, Non linear association

## Abstract

**Background:**

Many previous studies estimating the relationship between body mass index (BMI) and dental decay are conflicting. Most studies, however, examine the relationship using BMI as a categorical variable. This study evaluated the non-linear association between body mass index as a continuous variable and untreated dental decay.

**Methods:**

Cross-sectional study of adults free of diseases attending a tertiary dental clinic was conducted. The number of untreated caries at the time of consultation was assessed using the WHO criteria. A multivariable Poisson regression model for severity of untreated dental decay was first established. Restricted cubic spline functions were used to consider potential non-linear associations between BMI and untreated dental caries.

**Results:**

After multivariable adjustment, the prevalence ratios (PR) for the number of dental decay remained significantly associated with the age at beginning tooth brushing (PR = 1.15, 95% CI 1.05–1.25), BMI < normal (PR = 1.66, 95% CI 1.30–2.12), BMI > normal (PR = 1.30, 95% CI 1.03–1.65), SDI (PR = 0.61, 95% CI 0.50–0.75) and GI (PR = 1.59, 95% CI 1.30–1.94). When BMI was evaluated as continuous variable, it exhibited a significant U-shaped pattern with the number of untreated dental decay both in univariable and multivariable analysis.

**Conclusion:**

The rate of untreated tooth decay was associated with both under- and overweight status.

## Background

Both oral and general health status depends on a dynamic interplay, including the individual’s behaviour, particularly eating behaviours. Behavioural and lifestyle-related factors are significant contributors to dental caries initiation and development [1].

Despite improvements in preventive measures, dental caries remains highly prevalent, especially among specific groups. As a chronic disease, dental caries reflects the cumulative effect of harmful socio-economic, biological, and behavioural factors that occurred during the lifespan of a person [2, 3].

Based on the impact of dental health status on food choice [4–6], the association between dental caries and nutritional status was previously explored [7–10]. The exact underlying mechanism for the relationship is not clear. Untreated dental caries may cause changing eating patterns, but, eating patterns may increase the individual risk of developing more dental decay.

Until now, two hypotheses regarding the association between dental caries and BMI have been suggested: a positive association [5, 7–9] and a null association [10].

Most of these studies have used only a non-parametric approach [5, 7–10], by treating BMI as a categorical variable. However, Royston, et al. pointed out a number of disadvantages with this approach [11]. The most significant drawback is the loss of information and strength through what is equivalent to rounding.

Additionally, most studies have focused on adolescents and ignored the cumulative effect of unfavourable eating behaviour among adults.

Based on the assumption that untreated dental caries might decrease as well as increase energy intake, a U-curved association in which people with underweight and overweight report more untreated dental caries compared to people with a normal weight, may be expected. Therefore, the purpose of this study was to investigate whether a non-linear association may be expected between the number of untreated dental decay and BMI by maintaining BMI as continuous variable.

In addition, we examined whether socioeconomic factors may moderate this association among adults attending a tertiary health care centre in Morocco.

## Methods

### Study population

The present study included healthy patients older than 18 who were recruited from among adults seeking routine dental care at the school teaching hospital at Rabat Morocco. Eligible patients were volunteer adults with no history of other health conditions.

Adults treated for any other health condition or seeking the dental hospital for dental emergency needs were excluded.

The recruitment phase of the study was conducted between March and May 2012. Data in the present study were recorded from questionnaires, anthropometric measurements and oral examination.

### Definition of variables

Eligible adults were interviewed during their dental attendance. All interviews were conducted using a structured questionnaire designed for the present study, which included information on demographic data (age and gender), general health, education level, monthly family income and oral health behaviour.

Data were collected ensuring the privacy and confidentiality by face-to-face interviews and document review.

The monthly family income was measured relative to the Moroccan minimum wage during the period of data gathering. Three-point scales were used (low, moderate and high).

Educational level was divided into three categories: low (none or primary), average (secondary) and high (university education).

Oral health behaviours included information on frequency of tooth brushing and when the patients started tooth brushing. Frequency of tooth brushing was divided into brushing twice a day, more than twice a day and once or less a day. Brushing motion was also recorded as appropriate or inappropriate.

The nature of dental attendance was noted according to a two-category system (planned visit, acute visit).

### Anthropometric measurements

The interviewer measured the weight (kg) and height (m) at the time of interview and computed BMI. BMI was computed as weight divided by squared height (kg/m^2^).

Body weight and height were taken with participants in bare feet and light clothing, and measured to the nearest 0.1 kg and 0.1 cm, respectively. Body weight was measured using a portable digital scale and body height using a stadiometer.

### Oral examination

- Dental caries was recorded with tooth as the unit of measurement. The dental examination used international criteria standardized by the World Health Organization for oral health surveys [12]. The numbers of decayed (DT), filled (FT) and missing (MT) teeth were calculated. Dental caries diagnosis was based on visual-tactile criteria using a sterile mirror and a blunt dental probe. The examination was performed in a fully equipped dental clinic using plane mirror and sharp probe after the teeth had been dried with air. In this paper, the term “caries” includes caries with cavitation.
- Periodontal and oral hygiene status were assessed using the Community Periodontal Index (CPI), the Calculus Surface Index (CSI), the Gingival Index (GI) and the Simplified Debris Index (SDI).

Anthropometric measurements and oral examination were conducted by one examiner after calibration. Intra-examiner reliability was assessed by re-examining 10 volunteers after one week (Cohen’s kappa coefficient = 0.92).

### Statistical analysis

Data analysis was conducted using SPSS 13.0 (SPSS Inc., Chicago, IL, USA) and Stata 13 (StataCorp LP, College Station, TX, USA) software.

The data were presented as the mean ± standard deviation (SD) for continuous variable with a normal distribution, and as median with interquartile range (IQR) for variables with skewed distribution. For categorical variables, data were presented as proportion.

To evaluate the association between untreated dental caries and recorded variables, a Poisson regression analysis was first applied to estimate the Prevalence ratio (PR) of untreated dental caries through levels of various explanatory factors. The high prevalence of dental caries in the study group meant that odds ratios were poor indicators of relative frequency, so prevalence ratios were determined using Poisson regression modelling [13]. Prevalence ratio (PR) and related 95% confidence intervals (95% CI) were estimated in both univariate and multivariate regression analysis. The multiple regression model included the variables with p < 0.25. The adjusted rate ratios were considered statistically significant when p-values were 0.05 or less. The dependent variable used was the number of untreated decayed teeth (DT). The severity of dental caries at tooth level was defined as the increased amount of untreated dental decay. This means that we evaluated each subject’s likelihood to have more or less dental decay according to studied factors.

Three BMI categories were first used for analysis. Underweight subjects were defined as BMI < 18.5, normal weight as BMI of 18.5–24.99, and overweight and obese as BMI ≥ 25. Overweight and obese were combined during regression analysis due to the low proportion of obese subjects when analysing BMI as a categorical variable.

To avoid potentially arbitrary categorization, restricted cubic splines evaluated the likelihood of dental decay in each subjects according to the factors evaluated [13, 14]. Non-linearity was tested using the likelihood ratio, comparing the model with only the linear term to the model with both linear and cubic spline terms. We specified three knot positions at the 10th, 50th and 90th percentiles of BMI, that is, at BMI values of 17.75, 21.19 and 28.64, respectively. The reference level was set to the median value of BMI 23.23.

Poisson regression was used to compute prevalence ratios for the number of untreated dental caries, according to BMI. We controlled for age, income, frequency of tooth brushing, tooth brushing motion, age at beginning of tooth brushing, education level, simplified debris index, gingival index and reason for dental attendance.

## Results

A total of 101 adults volunteered to participate in the present study. The main characteristics of the subjects are summarized in Table 1.

**Table 1:** Characteristics of the study population

Variables	Values
Age (years)	27 ± 8
Female gender, n (%)	69 (68.31)
Income, n (%)	
Low	28 (27.73)
Medium	32 (31.68)
High	41 (40.59)
Frequency of tooth brushing, n (%)	
0 or 1/day	18 (17.83)
2/day	49 (48.51)
3/day	34 (33.66)
Tooth brushing motion, n (%)	
Appropriate	67 (66.34)
Inappropriate	34 (32.66)
Reason of the dental attendance, n (%)	
Planned visit	26 (25.74)
Acute visit	75 (74.26)
Education level, n (%)	
Non and primary	22 (21.78)
Secondary	29 (28.72)
University	50 (49.5)
BMI, n (%)	
Underweight	35 (34.65)
Normalweight	37 (36.63)
Overweight	22 (21.78)
Obese	7 (6.93)
Age at beginning tooth brushing (years), (mean ± SD)	3 ± 1
DT, (mean ± SD)	5.37 ± 3.66
MT, [median (IQR)]	4 (2–5)
FT, [median (IQR)]	2 (0–6)
DMFT, [median (IQR)]	12 (9–16)
GI, (mean ± SD)	1.42 ± 0.77
CPI, (mean ± SD)	1.15 ± 0.40
SDI, (mean ± SD)	1.28 ± 0.77
CSI, [median (IQR)]	1.26 (0.83–1.66)

*IQR* interquartile range, *SD* standard deviation, *F* female, *BMI* body mass index, *FT* number of filled teeth, *MT* number of missed teeth, *DT* number of decayed untreated teeth, *DMFT* number of decayed, missed and filled teeth, *GI* gingival index, *CSI* calculus surface index, *DIS* simplified Debris index, *CPI* community Periodontal index

The mean age of participants was 27 ± 8. Of the 101 participants, 69 (68.31%) were female. The BMI of 35 (34.65%) of subjects was below normal and the overall mean BMI was 22.31 ± 4.47.

The mean of teeth affected by dental caries was 5.37 ± 3.66, the median (IQR) of filled teeth was 2 (0–6).

Table 2 presents the results of Poisson regression analyses of the factors associated with the severity of dental caries at tooth level and the studied factors.

**Table 2:** Poisson regression analysis of the factors associated with the increased number of dental decay

	DT mean ± SD	Univariate analysis			Mulivariate analysis		
		PR	95% CI	p	PR	95% CI	p
Age (/year)		1.007	0.99–1.01	0.13	0.98	0.97–1.00	0,05
Gender							
Male	5.4 ± 3.7	1.03	0.86–1.24	0.68			
Female	5.2 ± 3.4	1					
Income							
Low	7.1 ± 3	1.65	1.30–2.07	<0.001	1.45	1.05–2.02	0.02
Medium	5.4 ± 3.5	1.25			1.06		0.62
High	4.3 ± 3.7	1	1.02–1.52	0.02	1	0.83–1.34	
Frequency of tooth brushing							
0 or 1/day	6.8 ± 3. 3	1.54	1.20–1.97	0.001	1.006	0.74–1.35	0.97
2/day	5.6 ± 3.6	1.34	1.06–1.58	0.009	1.03	0.83–1.28	0.74
3/day	4.2 ± 3.6	1			1		
Tooth brushing motion							
Appropriate	4.5 ± 3.5	0.65	0.54–0.77	<0.001	0.81	0.98–1.50	0.06
Inappropriate	7 ± 3.4	1			1		
Age at beginning tooth brushing		1.16	1.08–1.24	<0.001	1.15	1.05–1.25	0.02
Education level							
Non and primary	6 ± 3.6	1.35	1.09–1.68	0.006	1.14	0.81–1.58	0.43
Secondary	6.1 ± 3.4	1.49	1.23–1.81	<0.001	1.37	1.09–1.71	0.006
University	4.4 ± 3.5	1			1		
BMI							
Underweight	6.4 ± 3.9	1.58	1.29–1.94	<0.001	1.66	1.30–2.12	<0.001
Normal weight	4.4 ± 3.5	1			1		
Overweight and obese	5.3 ± 3.2	1.28	1.02–1.60	0.02	1.30	1.03–1.65	0.02
SDI	1.1 ± 0.3	0.88	0.79–0.99	0.03	0.61	0.50–0.75	<0.001
CSI*	0.5 (0.16–1.16)	0.94	0.84–1.07	0.40			
GI		1.19	1.06–1.33	0.002	1.59	1.30–1.94	<0.001
Reason of the dental attendance							
Planned visit	4.1 ± 3.3	0.73	0.54–0.90	0.004	0.94	0.74–1.18	0.62
Acute visit	5.7 ± 3.7	1			1		

* Median **(**interquartile range)

*SD* standard deviation, *F* female, *M* Male, *BMI* body mass index, *DT* number of decayed untreated teeth, *GI* gingival index, *CIS* calculus surface index, *SDI* simplified Debris index, *PR* prevalence ratio, *1* reference category

In univariate analysis, it was found that low and medium educational level (PR = 1.65, 95% CI 1.09–1.68; 1.49, 1.23–1.81 respectively), low and medium income (PR = 1.65, 95% CI 1.30–2.07; 1.25 95% CI 1.02–1.52), gingival index (GI) (PR = 1.19, 95% CI 1.06–1.33), age at beginning tooth brushing (PR = 1.16, 95% CI 1.08–1.24), underweight (PR = 1.58, 95% CI 1.29–1.94), overweight and obesity (1.28, 95% CI 1.02–1.60) frequency of tooth brushing (0 or 1/day and 2/day) were risk factors associated with the increased rate of untreated dental caries. At the same time, an appropriate motion of brush (PR = 0.65, 95% CI 0.54–0.77) and planned visit (PR = 0.73, 95% CI 0.54–0.94) were protective factors.

Adjusted multivariate analysis revealed that only age at beginning tooth brushing (PR = 1.15, 95% CI 1.05–1.25), BMI < normal (PR = 1.66, 95% CI 1.30–2.12), BMI > normal (PR = 1.30, 95% CI 1.03–1.65), SDI (PR = 0.61, 95% CI 0.50–0.75) and GI (PR = 1.59, 95% CI 1.30–1.94) remained associated with the increase of the number of dental caries.

**Fig. 1:**
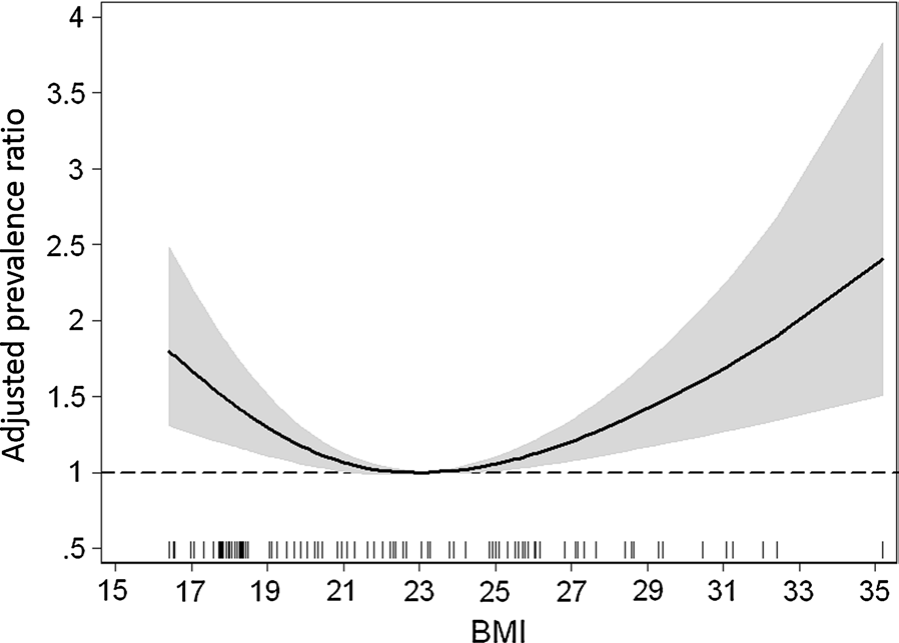
Relationship curves for association between the numbers of decayed untreated teeth and body mass index (BMI) on a continuous basis. Adjusted Prevalence Ratios are indicated by a solid line and 95% confidence intervals by gray color derived from restricted cubic spline with 3 knots

Figure 1 shows the adjusted rate ratios and confidence interval over the BMI values.

A significant quadratic effect between categorical BMI and the rate of untreated dental decay (the p values for non-linearity was <0.001 and for the overall effect p < 0.001) was observed.

This means that the number of untreated dental decay is a positive quadratic trend in the association with BMI values. So these data suggest a U-shaped trend in the association between untreated dental caries and BMI.

## Discussion

The present study indicated that adults with both low and high BMI have poorer dental health as manifested by more untreated caries compared with normal weight patients. This association was observed in both univariate and multivariate analyses and shown U-shaped distribution.

The association between unhealthy lifestyle and chronic disease continues to be a greater focus in public health because it is associated with substantial morbidity and mortality [15, 16].

Data from several population-based studies regarding the association between dental caries and BMI are conflicting. Patterns of previous reported relationships included no association [10] between BMI and dental caries, a positive association [17] and an inverse relationship [9, 18].

It should be stated that a majority of studies reporting positive association explored the association between obesity and dental caries [17, 19]. Most studies focus on linear (positive–negative) association.

Some studies suggest that dental caries is associated with both high and low body mass index [20, 21]. The present finding of a U-shaped association of BMI with dental caries suggest that this pattern of association is plausible.

We propose that association between BMI and dental caries may be explained thorough common explanatory factors. Unhealthy diets with frequent consumption of refined sugars and added sugars and low in fruit and vegetables may increase the level of both BMI [21] and dental caries. On the other hand, decreased food intake may be attributable to a high level of untreated dental caries and then explain frequency of untreated dental caries [9] among low-BMI adults.

BMI shares eating behaviours and social etiological factors with dental caries. Socio-economic factors are also indicators of individual behaviour regarding the types of food intake and may influence the dietary preference [22, 23].

Therefore, eating behaviours may mediate the link between BMI and dental health. Individuals with high risk should be encouraged to undertake lifestyle modifications and improvements in a dental health status diet.

An established relationship has been reported between brushing behaviour, educational level and the rate of dental caries. Findings of the present study corroborate these results [24].

Our results should be interpreted in light of both the study’s strengths and its limitations. High levels of dental decay were detected in the present study, which indicated a high-risk study population. Nonetheless, the associations detected may be generalizable to other study populations even if geographical inequalities in dental caries experience were reported [2, 24].

Unhealthy dietary patterns are prevalent among high-income countries and have emerged as public health conditions among low- and middle-income countries.

Such studies may then be crucial for estimating whether reductions in the prevalence of dental caries among specific groups may affect some populations’ morbidities like high and low BMI. Moreover, demand for better health systems throughout the world should integrate the possible co-existence of some pathologic conditions for common preventive measures.

Another limitation is the study design, which did not allow for assessment of temporality of associations. However, to our knowledge, this is the first study exploring the non-linear association between untreated dental caries and BMI as a categorical variable.

Future studies should explore more potential factors associated with unmet dental needs (dental insurance, barriers to access, psychological factors, etc.). In addition, dietary habits (composition and intake frequency) need to be considered.

In conclusion, a U-shaped trend in the association between dental decay and BMI was found. This means that an increased rate of untreated tooth decay was associated with both under- and overweight.

## Abbreviations

BMI: body mass index
PR: Prevalence ratios
SD: standard deviation
CI: confidence interval
Kg: kilograms
m: meter
